# Viral coinfection analysis using a MinHash toolkit

**DOI:** 10.1186/s12859-019-2918-y

**Published:** 2019-07-12

**Authors:** Eric T. Dawson, Sarah Wagner, David Roberson, Meredith Yeager, Joseph Boland, Erik Garrison, Stephen Chanock, Mark Schiffman, Tina Raine-Bennett, Thomas Lorey, Phillip E. Castle, Lisa Mirabello, Richard Durbin

**Affiliations:** 1 Division of Cancer Epidemiology and Genetics, National Cancer Institute, Rockville, Maryland USA; 2 Department of Genetics, University of Cambridge, Cambridge, UK; 3 Cancer Genomics Research Laboratory, Leidos Biomedical Research Inc., Frederick National Laboratory for Cancer Research, Frederick, MD USA; 4 Wellcome Sanger Institute, Wellcome Genome Campus, Hinxton, UK; 5 Women’s Health Research Institute, Kaiser Permanente Northern California, Oakland, California USA; 6 Regional Laboratory, Kaiser Permanente Northern California, Oakland, California USA; 7 Department of Epidemiology and Population Health, Albert Einstein College of Medicine, Bronx, New York USA

**Keywords:** HPV, Human papillomavirus, MinHash, Kmers, Coinfection, Bioinformatics

## Abstract

**Background:**

Human papillomavirus (HPV) is a common sexually transmitted infection associated with cervical cancer that frequently occurs as a coinfection of types and subtypes. Highly similar sublineages that show over 100-fold differences in cancer risk are not distinguishable in coinfections with current typing methods.

**Results:**

We describe an efficient set of computational tools, rkmh, for analyzing complex mixed infections of related viruses based on sequence data. rkmh makes extensive use of MinHash similarity measures, and includes utilities for removing host DNA and classifying reads by type, lineage, and sublineage. We show that rkmh is capable of assigning reads to their HPV type as well as HPV16 lineage and sublineages.

**Conclusions:**

Accurate read classification enables estimates of percent composition when there are multiple infecting lineages or sublineages. While we demonstrate rkmh for HPV with multiple sequencing technologies, it is also applicable to other mixtures of related sequences.

**Electronic supplementary material:**

The online version of this article (10.1186/s12859-019-2918-y) contains supplementary material, which is available to authorized users.

## Background

Human papillomavirus (HPV) is a DNA virus responsible for over half a million cervical cancer cases each year and an estimated 239,000 deaths worldwide [1]. Persistent infection with one of the carcinogenic HPV types is necessary for invasive cervical cancer development, and accounts for a large proportion of other anogenital and oropharyngeal cancers [2]. There are more than 200 papillomavirus types known to infect humans, with each type defined on the basis of at least 10% sequence difference in the L1 gene (major capsid protein) sequence. Not all HPV types contribute equally to infection or disease risk. Approximately a dozen of the more than 200 HPV types are considered carcinogenic, with just two types, HPV16 and HPV18, accounting for approximately 75% of cervical cancer cases worldwide [3].

HPV infection is not mutually exclusive to a specific type [4]. Concurrent infection with multiple HPV types is common, occurring in 20-50% of HPV infections [4–7]. One study reported nine distinct HPV types simultaneously in a single patient [8]. Co-infections appear to be random assortments of types with no evidence to support clustering of types or viral interactions between types [5].

Within each HPV type there are variant lineages which differ by 2-10%, and as little as 1% for sublineages, in their L1 gene sequence from other variants of the same type, and these also vary in risk for cervical precancer and cancer [9]. For HPV16, the most common and carcinogenic type, there are four main variant lineages (A, B, C, and D) and ten sublineages (A1, A2, A3, A4, B1, B2, C, D1, D2, and D3) that are roughly correlated with their geographic distribution. HPV16 sublineages show strong differences in histology-specific cervical precancer and cancer risks, with relative risks exceeding 100 for specific sublineages (D2, D3 and A4) associated with adenocarcinoma [10].

Mirabello et al. [10] used phylogenetic methods and lineage-specific SNP genotyping to detect HPV16 lineages. While able to accurately determine the dominant lineage, Mirabello et al. were not able to assess whether samples were infected with multiple lineages. There is little known about the epidemiology of co-infections with multiple HPV16 variant lineages, though this is clinically relevant given the significant differences in risk associated with each lineage.

Here we present a toolkit, rkmh, developed to help characterize HPV coinfections at the type and lineage level. Our toolkit makes use of the MinHash locality-sensitive hashing scheme, a technique developed for detecting similarity in webpag es that has been previously applied in metagenomics [11]. Tools are included for classifying reads and removing contaminating sequences. A pipeline specifically for analyzing HPV16 lineage coinfections is also included. rkmh is written in C++ and can classify a deep-sequenced HPV16 sample in minutes on a laptop computer. While applied here to HPV, the tools in rkmh are data agnostic and could be applied to other genomes of interest and read technologies without requiring any modifications.

## Implementation

We developed rkmh based on methods introduced in [11], extending their algorithm to use various filters at the per-read level which improve classification performance. We also maintain information about type and lineage assignment on a per-read basis to enable estimation of relative abundances in a mixed infection.

rkmh is written in C++ and is threaded with OpenMP. It is freely available under the MIT open source software license at github.com/edawson/rkmh.

### Hashing reads with rkmh

Much like Mash [11] and sourmash [12], rkmh relies on MinHash to transform reads for similarity comparison. Briefly, the algorithm works by generating all consecutive overlapping kmers of the read and hashing them with MurmurHash3 (Austin Appleby, https://github.com/aappleby/smhasher) to 64-bit integers. These integers are then sorted. A subset of size *N* of these hashes, usually the lowest *N* according to standard numerical ordering, are then chosen as a signature or ’sketch’ of the read. This effectively represents a sample of the kmers present in a read. MinHash is locality-sensitive at the sketch level: reads which are more similar will share more kmers. By comparing only *N* integers, the number of comparisons per reference is reduced by *L*−*k*−*N* where *L* is the length of the genome and *k* is the kmer size.

### Classifying reads

Reads are classified by first generating the MinHash sketches for the reference sequences. A MinHash sketch is then generated for each read. All sketches use a single, fixed kmer size *k* and sketch size *N*. Abundance and uniqueness filters are optionally applied at this stage. Each read’s sketch is then compared to each reference sketch. The intersection of the two sketches is calculated in *O*(*N*) time where *N* is the sketch size. The read is then labeled as the reference with which the read shares the largest number of hashes.

### Filtering kmers to improve classifications of individual reads

To improve specificity we implemented a set of kmer- and read-level filters in rkmh that are not offered by other MinHash-based classifiers. The classify, stream, and filter commands support four filters. The first is a floor for kmer abundance in reads (−*M*). As the reads are hashed we store the number of times each hash is seen. Any hashes that do not meet the threshold for abundance are then excluded from a read’s MinHash sketch. [11] implemented this filter to remove sequencing errors in sketches of read sets; here we have simply extended it to remove them in individual read sketches. The second available filter is a ceiling on the number of times a hash may occur in the reference sequence set (−*I*). This filter is designed to remove repetitive kmers or those shared among many references, making them uninformative. We also implement a minimum difference filter (−*D*) that flags read sketches if the difference between the first- and second-best classifications is less than the desired threshold. This removes reads that cannot be given a unique classification because they come from genomic regions shared among references. Finally, a minimum number of shared hashes may be set so that reads that do not match well to any reference are flagged (−*N*).

### Filtering reads

We initially tried assessing the performance of our type classifier on raw data but found that its performance was very poor, with high rates of supposedly false negatives. We performed a BLASTN [13] search on some of these reads to find that many of their top hits were in the human genome. We implemented a filter to deal with this at the classification level but realized that such a feature would also be useful in filtering a FASTQ file to find only reads which come from the organism of interest. The rkmh filter command implements the filters used in classification to filter reads. The rkmh stream command also implements an option for this, allowing real-time filtering of FASTQ reads during analysis.

### Quantifying lineage and sublineage prevalence within a sample

Lineage and sublineage strains are differentiated mostly by SNVs and small INDELs. These polymorphisms alter the kmers of the sequence. If these kmers are unique among the reference sequence they can be used as a way of quantifying the strain they define. We implement an exact kmer matching strategy in rkmh by removing all kmers that appear in multiple references. This creates a minimal sketch that contains kmers unique to each reference sequence. Each read is kmerized, hashed, and then compared against these reduced sketches. Reads that match well to a given reference sketch can be used to estimate the reference strain’s abundance in that set of reads. This process has been wrapped in the rkmhhpv16 command. When run in the rkmh directory, all reads in a fastq file can be labeled with their HPV type and HPV16 lineage/sublineage by running:


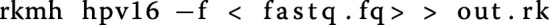


The read classifications can be converted to lineage/sublineage prevalence estimates by running:


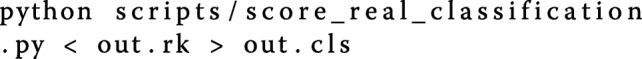


This will produce a file that contains a single line listing the estimated lineage and sublineage frequencies.

### rkmh output formats

There are three main output formats produced by rkmh. The outputs of the stream and classify commands are a tab-separated classification description similar to that produced by [11]. This format is easily manipulated using command line tools such as grep, cut, and sed, making analysis on any Unix system simple and portable. Additionally, the rkmh hash command can output sketches in JSON or the vowpal-wabbit vector format, a tab-separated format used by the vowpal-wabbit machine learning package [14]. The version used by rkmh needs only to be labeled with its correct class by replacing a single sentinel string using sed. Sketches and vw-vectors may be computed for individual reads in a FASTA/FASTQ file or for the entire file.

### Generation of simulated data

To assess the performance of rkmh we generated simulated read sets of coinfected and non-coinfected samples at known mixture proportions. We simulated reads at extremely high depth from 62 manually-prepared HPV16 sublineage reference genomes using DWGSIM (Nils Homer, https://github.com/nh13/DWGSIM). We set DWGSIM to create 225 basepair reads using the Ion Torrent error profile and flow order. This produced a set of large FASTQ files, one for each sublineage. We generated random coinfections using the scripts at https://github.com/edawson/siminf. Briefly, siminf randomly selects an overall coverage to simulate along with a list of infecting strains and their relative proportion. A minimum of 5% strain abundance is required. siminf then samples our large sublineage FASTQ files to generate a FASTQ containing reads from the chosen sublineages in the desired proportions. We provide 50 of these simulated coinfections in https://github.com/edawson/rkmh_sim_data; more can be generated using the siminf package or by request.

## Results

### HPV typing performance across sequencing technologies is sensitive to kmer and sketch size

We assessed the HPV typing performance of rkmh on three datasets: simulated 100bp paired end Illumina reads based on the PAVE database of HPV reference genomes [15]; a real HPV16 sample sequenced on the Ion Torrent Proton platform (typical read length 250bp); and a set of 3660 Oxford Nanopore minION reads generated from two HPV16 reference strains (typical read length over 6500bp). The minION reads typically cover the majority of the 7-8kb HPV genome, but have a relatively high error rate of 10% or more, comparable to the difference between HPV types and greater than that between lineages (they were collected in 2015 using the R7 pore).

**Fig. 1:**
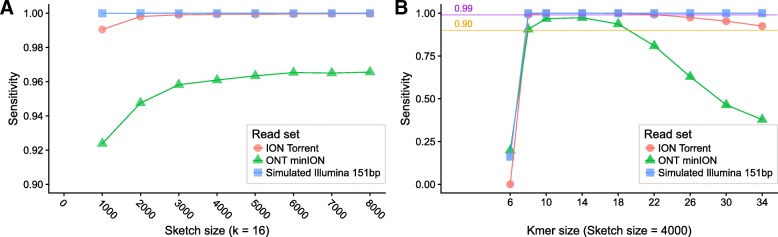
Sensitivity of rkmh with respect to sketch size (**a**) and kmer size (**b**). There are diminishing returns to increasing sketch size above roughly 4000, regardless of read length. (**b**) shows that kmers are not sufficiently unique to classify reads with k ≤10. Above k = 18, sensitivity begins to drop, likely due to the effects of incorporating sequencing errors into kmers. This is especially noticeable for ONT minION reads, which have a much higher error rate (above 12% per base for the R7.4 pore) compared to ION Torrent and Illumina (<0.1*%* per base)

MinHash-based methods depend on a “sketch” which is a characteristic subset of kmers from a set of input sequences. Even at a low sketch size of 1000, rkmh correctly classifies more than 99% of the short reads and more than 90% of the nanopore reads (Fig. 1a). As sketch size increases to 4000, per-read accuracy approaches 100% for short reads and 96% for ONT minION reads, with negligible improvements for sketch sizes higher than 4000. Sketch sizes below 1000 are not sufficiently sensitive for classifying HPV types, showing per-read accuracies well below 90%.

Kmer size is the main determinant of MinHash classification performance when errors are present. For HPV type classification we find that performance is diminished above k = 18 for our Ion Torrent reads and above k = 14 for our ONT minION reads (Fig. 1b). This is due to the introduction of kmers containing one or more sequencing errors. The high per-base error rate of the ONT minION R7.4 pore (12% total per base [16]) means that as kmer size increases there is a rapid accumulation of kmers that do not match the reference because of incorporated errors, to the extent that for some reads no diagnostic kmer is found.

We compared the performance of rkmh to Taxonomer [17], a tool commonly used for metagenomic classification but which is not specifically designed for viral classification. On the set of 3660 HPV16 minION reads, Taxonomer reported that 42.4% were of viral origin and 8.3% were from HPV16. It also reported 1177 bacterial reads and 304 human reads; 398 reads were unclassified. rkmh reported 3381 (92.4%) as HPV16. When we ran Taxonomer on a simulated 250bp ION Torrent HPV16 coinfection data set (discussed further below), it reported that 29.2% of reads were HPV16, whereas rkmh reported that 94% of reads came from HPV16. In summary, Taxonomer has substantially lower sensitivity and specificity than rkmh for this type of data and analysis – this is not surprising since taxonomer is a general purpose metagenomics classification tool, which is not designed for medium to long read length viral sequence analysis.

### Kmer pruning improves classification performance

**Fig. 2:**
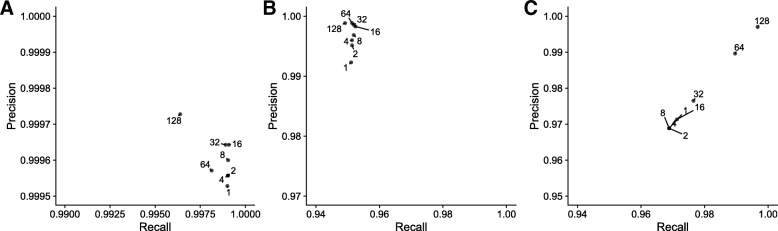
Precision/recall plots for type classification of 70,000 Ion Torrent reads from an HPV16 amplicon sequencing reaction (**a**) and 3660 ONT minION reads derived from two HPV16 isolates (**b**, **c**) at various read sketch pruning levels *M* indicated by the label attached to each point. Read sketch pruning removes rare kmers in the read sketch which might be random sequencing errors. (**a**, **b**) were classified using a kmer size of 16 and (**c**) was classified using a kmer size of 10. Ion Torrent reads have low substitution error rates, so pruning removes few kmers and the precision boost is small (<0.001%) (**a**). ONT minION reads have a much higher error rate approaching 10% per-base. For minION reads, pruning is able to improve precision to roughly 99.8% when using a kmer size of 16 (**b**). A smaller kmer size of 10 combined with high levels of pruning lead to an increase in both precision and recall, with precision and recall increasing from slightly more than 97.0% to over 99% (**c**)

We can increase the type classification rate for minION reads by decreasing the kmer size at the cost of introducing false positive assignments to other HPV types. However, this effect can be counteracted by removing kmers that are rare in the read set or enriching for those that distinguish between reference genomes. Such filters have been previously applied across read sets but not for individual reads. We term this sketch modification process “pruning” and describe the individual filters in more detail in the “Implementation” section. Figure 2 shows the effect of pruning readset kmers on the ability of rkmh to classify Ion Torrent and minION reads. Increasing read pruning via the *M* parameter has a negligible effect on Ion Torrent reads as they have a low error rate (<<1*%*) and are relatively short; the majority of information available in them is acquired using just the default rkmh settings. MinION reads, while possessing a higher error rate, also possess many more kmers, meaning that dropping an erroneous kmer from the read sketch makes room for a possibly informative one. By dropping the kmer size from *k*=16 to *k*=10 and increasing the readset pruning threshold, we improve both precision and recall of our read classification by roughly 2% (Fig. 2c).

These results demonstrate that rkmh is suitable for HPV typing. More than 90% of the individual reads match their known correct HPV type across Ion Torrent, ONT minION, and simulated Illumina datasets. Kmer pruning can further improve classification performance for long, noisy reads. From these per-read classifications one can determine the proportions of the infecting types by tallying the number of reads that support each type.

### Accurate read classifications enable accurate percent composition estimates of HPV types

**Fig. 3:**
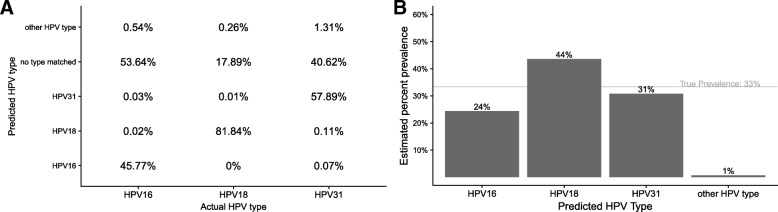
**a** The performance of rkmh on a simulated HPV type coinfection. Summing the rows of this matrix gives percent prevalence estimates for each type **b**

We next simulated a coinfection of HPV16, 18, and 31 by combining at equal proportions Ion Torrent reads from known samples of a single HPV type. We also examined the same sample after removing reads which did not map to the HPV genome(s), of which there are many (Fig. 3a). We summed the number of reads classified by rkmh to each HPV type with more than 5 kmers and divided each sum by the total number of reads classified to estimate the percent prevalence. rkmh is able to detect all three HPV types, though their proportions are off by 5-15% (Fig. 3b). Most of the reads are unclassified. We expect many of the unclassified reads may contain bits of human sequence and that our HPV18 sample appears over-reported simply because it had the most HPV DNA of the three. When restricting to reads that map to the HPV16, HPV18 or HPV31 genomes, rkmh accurately classifies over 99% of the reads into the correct type at the default settings (Additional file 1: Figure 1). rkmh produces essentially perfect estimates of percent composition on this filtered subset.

We then applied rkmh to ten real samples amplified using a universal HPV primer scheme, sequenced on the ION Torrent and annotated with infecting HPV types by manual review. In eight out of the ten samples, rkmh correctly identifies all of the manually annotated types using the default parameters (k = 16, s = 1000, threshold ≥1*%* or ≥1000 reads) (Additional file 1: Table 1). Both the two samples where the classifications differ involved marginal decisions. For one sample a type that had not been previously annotated was reported with 1.4% of reads assigned to it. For another sample a previously annotated type only received 942 reads, just below our reporting threshold of 1000. This was still more than 20 times more than the next highest type (41 reads), so could have been examined as a borderline case without generating noise. Based on the performance of rkmh on both our simulated set and our ten real samples, we believe it is providing reliable type estimates in line with previous annotations.

### Classification and quantification of HPV16 lineage coinfections

**Fig. 4:**
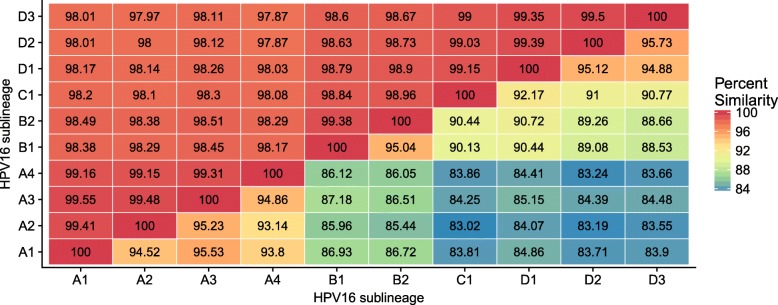
Percent similarity for HPV sublineage; numbers above the diagonal are nucleotide similarity. Numbers under the diagonal are similarity estimates based on the number of shared hashes from rkmh

HPV16 lineages and sublineages differ by less than 10% of L1 sequence. HPV16A and HPV16D differ the most among HPV16’s lineages but still share more than 97% identity. Within the A lineage the A1, A2, A3, and A4 sublineages differ by less than 1% (Fig. 4). MinHash similarity estimates and nucleotide similarity are highly correlated (*r*=0.9947), but MinHash estimates show a bigger spread than nucleotide similarity because a single base change affects the *k* adjacent kmers. In essence, MinHash (and kmer-based methods in general) exaggerate differences between sequences, compared to direct string comparison.

**Fig. 5:**
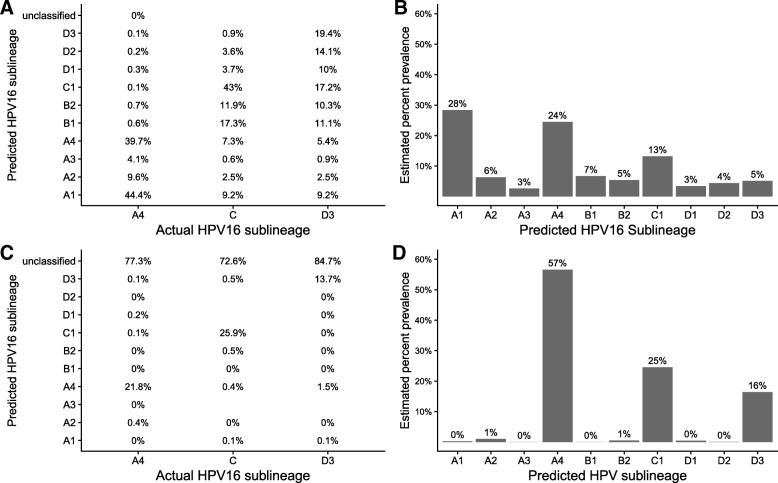
**A** The percentage of reads from a simulated coinfection classified by rkmh to each of the HPV16 sublineages, at default settings (k = 16, s = 1000, no pruning, no difference filter). Summing each row of **a**, with the exception of reads that couldn’t be classified, gives the percent prevalence estimate of each sublineage (**b**). **c** The percent of reads classified to each sublineage by rkmh at pruning level M = 100 and I = 1. This significantly improves the prevalence estimates (**d**)

To assess rkmh’s ability to discriminate coinfecting lineages using sketch pruning, we simulated a coinfection of HPV16 A4 / C / D3 in a 54:26:20 ratio. We show the per read performance (Fig. 5a) as well as rkmh’s estimated percent composition of our sample (Fig. 5b) at various parameterizations. At the default settings (i.e. the standard MinHash algorithm, k = 16, s = 1000) there is a large amount of noise in the lineage classifications and the estimated percent compositions are similarly affected. Sublineage A1 is estimated to be the dominant sublineage even though no reads from sublineage A1 are present.

We applied sketch pruning to remove kmers that are shared among sublineages, adding a parameter *I* that removes kmers seen in more than *I* references (see Implementation). At I = 1 each kmer in a reference sketch will be unique to a single sublineage. This effectively removes shared portions of the genome and reduces the MinHash procedure to exact kmer matching. Raising the pruning level to I = 1 is sufficient to reduce erroneous read classifications from approximately 30% of reads misclassified to less than 5%; this comes at the expense of 60-90% of reads from each sublineage being removed from analysis (Fig. 5c). This leads to much better estimates of sublineage prevalence (Fig. 5d). Pruning is more effective at removing false classifications than simply requiring a minimum number of differences between a read’s two best classifications (a filter implemented in other MinHash packages) (*s*=8000,*D*=20; not shown). Sketch pruning at *I*=1 does not meaningfully affect type classification (not shown). For the HPV16 specific workflow, we use the set differences of sublineage hashes to strictly remove kmers that appear across multiple sublineages. This enforces that each kmer appears in only one sublineage sketch; this provides only a minor improvement over the standard pruning implementation (Additional file 1: Figure 2), which is much faster. These results are representative of repeated tests on simulated coinfections (data available at https://github.com/edawson/rkmh_sim_data), and we find that the overall correlation between rkmh estimated prevalence and the true sublineage prevalence is 0.95.

We next performed a systematic analysis of the effects of divergence, read length, and error rate on read classification performance. We simulated three lineage references A, B, C with random divergence rates 0.5%, 1%, 2.5% from the HPV reference. Then we simulated 3 sublineages A1, A2, A3, B1, B2 etc. at random divergence distances 0.05%, 0.1%, 0.25% from each of their lineage references. Then for each reference set we simulated a million reads, selected evenly from these sublineages for each of the following sequence models, chosen to reflect the range of different read lengths and error rates available in practice: 75bp 0.1% error (short Illumina) 150bp 0.5% error (long Illumina) 250bp 1% error (IonTorrent) 5000bp 10% error (long read single pass) 5000bp 1% error (long read multi-pass) The design of three potential references at both lineage and sublineage level allowed us to evaluate false positive rates in terms of assignment to the lineage and sublineage not present in the data, as well as sensitivity in terms of correct assignment. For reads 250bp or longer, we found that >80% of reads were correctly classified to their known lineage and pruning could reduce false positive assignments to almost zero (Additional file 1: Figure 3). We therefore expect rkmh to produce accurate lineage quantifications for ION Torrent data. At the sublineage level, we found that rkmh performed poorly at default parameters across read types (as expected) but that kmer pruning could reduced the false-positive sublineage assignments to less than 0.1*%* of reads (Additional file 1: Figure 4). Sublineage sensitivity was largely determined by divergence from the reference, with two-fold differences in the percentage of reads correctly classified between 0.05% and 0.25% divergence. While this can bias estimated proportions for sublineages, individual read classifications using kmer pruning are highly specific, indicating that rkmh can still detect the presence or absence of sublineages based on the presence of high-confidence read assignments.

Since rkmh can characterize simulated coinfections adequately, we assessed its performance on real coinfections identified in samples from Mirabello et al. 2016 [10]. In roughly 90% of real cases we examined rkmh agreed with the manually annotated predominant infecting lineage and sublineage (Table 1). We also find good concordance (70% or more) with manual annotations for coinfection status, where we consider a sample coinfected if a second lineages/sublineage is represented in at least 1% of reads. We can identify a coinfected secondary lineage with similar accuracy. However, our performance on identifying any secondary sublineage(s) is only 35%. Further review of samples for which rkmh did not agree with the manual annotations indicated that many had characteristics which make them difficult or impossible to correctly classify. In some samples, the two dominant sublineages had frequencies that were close to equal and rkmh correctly predicted the infecting sublineages but not their order. When a sample possessed a sublineage not in the reference set, rkmh often predicted the correct lineage but assigned reads evenly among the sublineages in the family. This sometimes falsely indicated a coinfection was present at the sublineage level. Lastly, a small proportion of samples we examined were of low coverage or quality and had no reads that could be used for classification.

**Table 1:** Performance of rkmh on samples from [10] which were manually reviewed for their infecting sublineages and coinfection status

N = 34 manually annotated samples	Agrees with annotations	disagrees with annotation	Concordance
Primary Lineage	32	2	95%
Primary Sublineage	31	3	91%
Secondary Lineage	24	10	71%
Secondary Sublineage	12	22	35%
Coinfection status, lineage	27	7	79%
Coinfection status, sublineage	24	10	70%

### Run time performance of rkmh

rkmh was designed to scale to millions of reads and genomes megabases in size. Classifying over 400,000 Ion Torrent reads against all 182 HPV type references in PAVE requires less than one gigabyte of RAM and runs on a quad-core Intel desktop in 1 min 16 s. In general, rkmh can process around 250,000 basepairs per core-second and scales well to increasing numbers of cores. Run times are dominated by sketch size and the number of reads as these two parameters affect the total number of comparisons to be made. Memory usage is dominated by the size and number of the reference genomes, meaning that there is not a major penalty for using long reads and that memory usage remains relatively constant over time. We have tested rkmh on ONT minION reads from genomes as large as 4.5 Mbp (*Escherichia coli* strain K-12) in under 16 GB of RAM using sketch sizes in the tens of thousands (data not shown).

## Discussion

There are various factors that can lead to biases or incompleteness in the application of rkmh. In our unique kmer matching sketches, each sublineage is defined by between 145 and 440 unique kmers. HPV sublineages with more available unique kmers may be more detectable, biasing results toward more divergent sublineages. It is also important to note that the amplicon sequencing scheme used to sequence the Ion Torrent samples does not produce consistent depth across the genome. If mutations are not randomly distributed, and regions of diversity are not evenly sequenced, this difference in depth could reduce the correlation between kmer prevalence and strain prevalence. All our data were produced by amplicon approaches, so should not include fusions with host DNA; however if such sequences were present due to other enrichment approaches they might increase noise and reduce signal for some reads but should not lead to biases, assuming multiple integration sites. Long reads from single-molecule sequencing should provide more specific per-read classifications and therefore better estimates of sublineage prevalence once the technology becomes cost efficient. MinHash, while a viable method when strain prevalences are high, may not be a viable estimator of very low-prevalence (≤5%) coinfecting lineages and sublineages.

We may not expect all HPV16 sublineage isolates to perfectly match our reference genomes as the virus continues to evolve, albeit slowly. Many of our secondary sublineage classifications which we label “incorrect” may well be isolates harboring mutations present in multiple sublineages. This highlights the fact that our classifications are only as good as our reference panel. In an early run of our pipeline we mistakenly left out the sequence for sublineage A2, and this had a significant impact on our sensitivity for non-A lineage reads as many reads were discarded in A2-infected samples. The upside of this is that future domain knowledge may yield even better classifications.

We also note that our reference set is based on annotations that were performed by hand in IGV and may contain mistakes and differences in opinion. In particular, some of our errors at the level of secondary lineage/sublineage may be affected by variation in reference classification. As each read is independently classified we believe this may indicate that some of our samples require further manual review.

With respect to possible future improvements to rkmh, Ondov et al. discuss possible performance improvements to the MinHash scheme in [11]. Sequence Bloom Trees are data structures that would allow MinHash sketch comparison in logarithmic rather than linear time. An alternative to the Sequence Bloom Tree would be to use the minimizer database described in [18] to assign genus-level labels to reads in metagenomic samples, though the kmer sizes we use for HPV16 classification may be too small to make this sensible. Additionally, many existing packages support pre-hashing sequences, which amortizes the expense of this procedure over later comparisons. rkmh will implement this in a future release. rkmh also removes the p-value defined in [11], which becomes harder to interpret on a per-read basis and which is affected in complex ways by the various filters in rkmh.

Several modifications to the sketching procedure might improve classification performance. Skip-grams (kmers generated from genomic substrings length $\frac {k}{2}$ separated by a small, fixed distance) would improve classification if genomes share rearrangement patterns. Using minimizers, where sketches are composed of hashes sampled from rolling genomic windows (rather than randomly sampling the entire sequence as in MinHash) would provide more even coverage of the reference sequences, possibly improving the chances of a read matching. Dynamic sketch sizes based on the length of the query sequence (rather than a fixed sketch size) might provide a slight improvement in runtime. Classification might be improved by introducing machine learning techniques trained on full sketches, as our supervised approach may overlook cryptic but important features. Finally, we believe that an improvement in data quality from long, high-quality reads will yield a large improvement in results when such data becomes available, and could be instrumental in advancing scientific inquiry and eventually developing effective public health measures to address HPV infection.

## Conclusions

HPV is a common sexually-transmitted agent, and a small subset of HPV infections become chronic and can lead to cervical, anogenital or oropharyngeal cancer. Twelve of at least 170 known HPV viral types are currently associated with cancer risk, and sublineages within these carcinogenic types are further associated with variable risks. Confounding proper classification of HPV infections is the prevalence of multiple types, lineages, and sublineages in individual infections. Thus, the accurate detection of HPV types, as well as HPV16 lineages and sublineages, could have important pleiotropic implications for public health measures.

We developed a computational toolkit to classify coinfected HPV samples, as in [10]. Our method, rkmh, is a collection of tools that addresses some of the challenges associated with analyzing mixtures of biological sequences. To implement rkmh we extended existing work utilizing the MinHash locality-sensitive hashing scheme [11], resulting in a tool that provides accurate classifications of individual reads. Accurate classification of the infecting viral types, lineages and sublineages is critical given the vast differences in disease risk between HPV types and even closely related HPV16 sublineages. Our toolset demonstrates that accurate classification of individual reads and estimation of type and lineage prevalence is possible with current sequencing practices, but that sensitive sublineage detection may require improvements in technique.

While applied here to HPV, rkmh could be used in any context where quantification of specific sequences within a mixture and selection for or removal of such sequences might be useful. MinHash has previously been applied to larger metagenomic datasets with striking success. Ondov et al. demonstrate MinHash’s ability to work on genomes several megabases in size and scale to billions of reads in [11]. Other viruses show significantly more intra-host variation than HPV; notably, Human Immunodeficiency Virus (HIV) evolves during infection and in response to treatment [19]. Zika and Ebola are urgent public health threats, have been shown to evolve over the course of outbreaks, and have been successfully sequenced in the field on the ONT minION [20–22]. The ability to generate per-read classifications using rkmh on a standard laptop could be a useful addition to the current pipelines employed by these studies. Lightweight algorithms such as rkmh may also be of interest in areas with strict computing power limitations such as space genomics.

## Additional file

Additional file 1 This contains supplementary figures 1 to 4 and supplementary table 1 (docx 147 kb)

## Data Availability

*Project name:* rkmh *Project home page:* https://github.com/edawson/rkmh *Operating system(s):* Unix including Linux and MacOS *Other requirements:* Python, gcc, zlib, OpenMP *License:* MIT *No restrictions on use by non-academics.* The simulated data sets used in this study are available in Github https://github.com/edawson/rkmh_sim_data.

## Abbreviations

HIV: Human immunodeficiency virus
HPV: Human papilloma virus
