# Supplementary material for: Viral coinfection analysis using a MinHash toolkit
Source: BMC Bioinformatics. 2019 Jul 12;20:389. doi: 10.1186/s12859-019-2918-y (PMC6626348; doi:10.1186/s12859-019-2918-y)
Supplement: Supplementary file 1 — This contains supplementary figures 1 to 4 and supplementary table 1 (docx 147 kb) [file 12859_2019_2918_MOESM1_ESM.docx]

Supplementary Figure 1: Type classification of real HPV16, HPV18 and HPV18 reads with MAPQ >= 30. These were sampled and mixed in equal parts (1/3 (700 reads each for a total of 2100 reads). (A) Per-read classification rates for all types exceeds 99%; one HPV31 reads is misclassified. Type prevalence estimates exactly match the true prevalence (B).

Supplementary Figure 2: Sublineage classification of simulated reads and corresponding prevalence estimates using different rkmh runtime parameters. (A) Per-read classification rates at the default settings (s = 1000, k = 16, no pruning) are poor, with many off-target matches. (B) This is reflected in the prevalence estimates, where a high proportion of sublineage A1 is reported though no A1 reads were present. (C, D) Read classifications and prevalence estimates at (s = 8000, k = 16, I = 1 and M = 5) are significantly improved, though still somewhat noisy. (E, F) Performance at (s = 8000, k = 16, I = 1, M = 100) is further improved; pruning of the read sketches leads to better read classifications. (G, H) Classifications using the hpv16 pipeline, which is equivalent (s = 8000, k = 16, I = 1, M = 5) but using a strategy that removes all kmers shared across references, rather than the approximate technique used by the sketch command.

Supplementary Figure 3: Per-read lineage classification performance on different simulated HPV16 sublineage read sets. Lineage classification performance increases with read length and divergence. A: Short (75bp) Illumina reads show the worst classification performance, likely because a read may not be long enough to capture a lineage-defining SNP. However false-positive assignments are almost completely removed using kmer pruning (I=1). B and C: Performance on 150bp and 250bp reads is much better, with most false positives removed at 250bp even without kmer pruning. D and E: For 5000bp reads, rkmh is 100% accurate at lineage classifications across the spectrum of genome divergence and error rates.

Supplementary Figure 4: Per read sublineage classifications for simulated HPV16 sublineage reads. Panels as in Supplementary Figure 3. In the absence of kmer pruning (I=1), false positive levels are high for short reads. This problem is fixed with kmer pruning but sensitivity is then low unless long reads are used (D and E), and even then increased sketch size (parameter s) can be important to achieve high sensitivity.

| Sample | Annotated HPV types | Types identified by rkmh (> 1% of reads or >1000 reads)  (s = 1000, k = 16) | % of reads identified |
| --- | --- | --- | --- |
| HPV16_1507 | 16 | 16 | 99.9% |
| HPV16_1780 | 16 | 16 | 99.9% |
| HPV33_PAP221833 | 33 | 33 | 99.9% |
| HPV58_1134 | 58, 16 | 58, 16, 33 | 57.0%, 38.0%, 1.4% |
| HPVco1633395859_1435 | 16, 33, 39, 58, 59 | 39, 16, 59, 58, 33 | 78.7%, 10.8%, 8.2%, 1.3%, 0.5% |
| HPVco1633_1488 | 16, 33 | 33, 16 | 98.5%, 1.4% |
| HPVco185168b_1708 | 18, 51, 68b | 18, 51, 68 | 53.0%, 44.2%, 2.4% |
| HPVco4551_1190 | 16, 45 | 45, 16 | 96.7%, 3.0% |
| HPVco1639525658_1261 | 16, 39, 52, 56, 58 | 56, 16, 52, 39, 58 | 28.5%, 31.3%, 20.3%, 11.1%, 8.5% |
| HPV52_PAP208646 | 52, 16 | 52 | 99.9% |

Supplementary Table 1: rkmh estimates for HPV type prevalence in ten samples that were amplified on a universal HPV primer panel, sequenced on the ION Torrent, and annotated for infecting types at NCI. rkmh predicts the manually reviewed annotated types above 1% prevalence in 8 of 10 samples. In sample HPVco1633395859_1435, type 33 is reported by rkmh at 1.4% prevalence, but this was not reported in the manually reviewed annotation. In sample HPV52_PAP208646, HPV16 was listed in the manually reviewed annotation but reported by rkmh in only 942 reads (0.08%), just below the default reporting threshold for rkmh.
